# Incidence of SARS-CoV-2 Infection during the Omicron Variant Emergence in Southern Vietnam: Prior Infection versus Third-Dose Vaccination

**DOI:** 10.1128/spectrum.01175-22

**Published:** 2022-08-24

**Authors:** Thang Thanh Phan, Tran Bao Nguyen, Quynh-Giao Thi Phung, Vinh Thanh Tran, Toan Trong Ho, Suong Phuoc Pho, Trung Hien Quach, An Thuy Truong, Hang Thuy Nguyen, Thuc Tri Nguyen, Son Truong Nguyen

**Affiliations:** a The Laboratory D Unit, Cho Ray Hospital, Ho Chi Minh City, Vietnam; b Department of General Director, Cho Ray Hospital, Ho Chi Minh City, Vietnam; c Department of the Vice-minister, Ministry of Health, Hanoi City, Vietnam; Pontificia Universidad Católica de Chile

**Keywords:** SARS-CoV-2, Omicron, reinfection, third-dose vaccination

## LETTER

**FIG 1:**
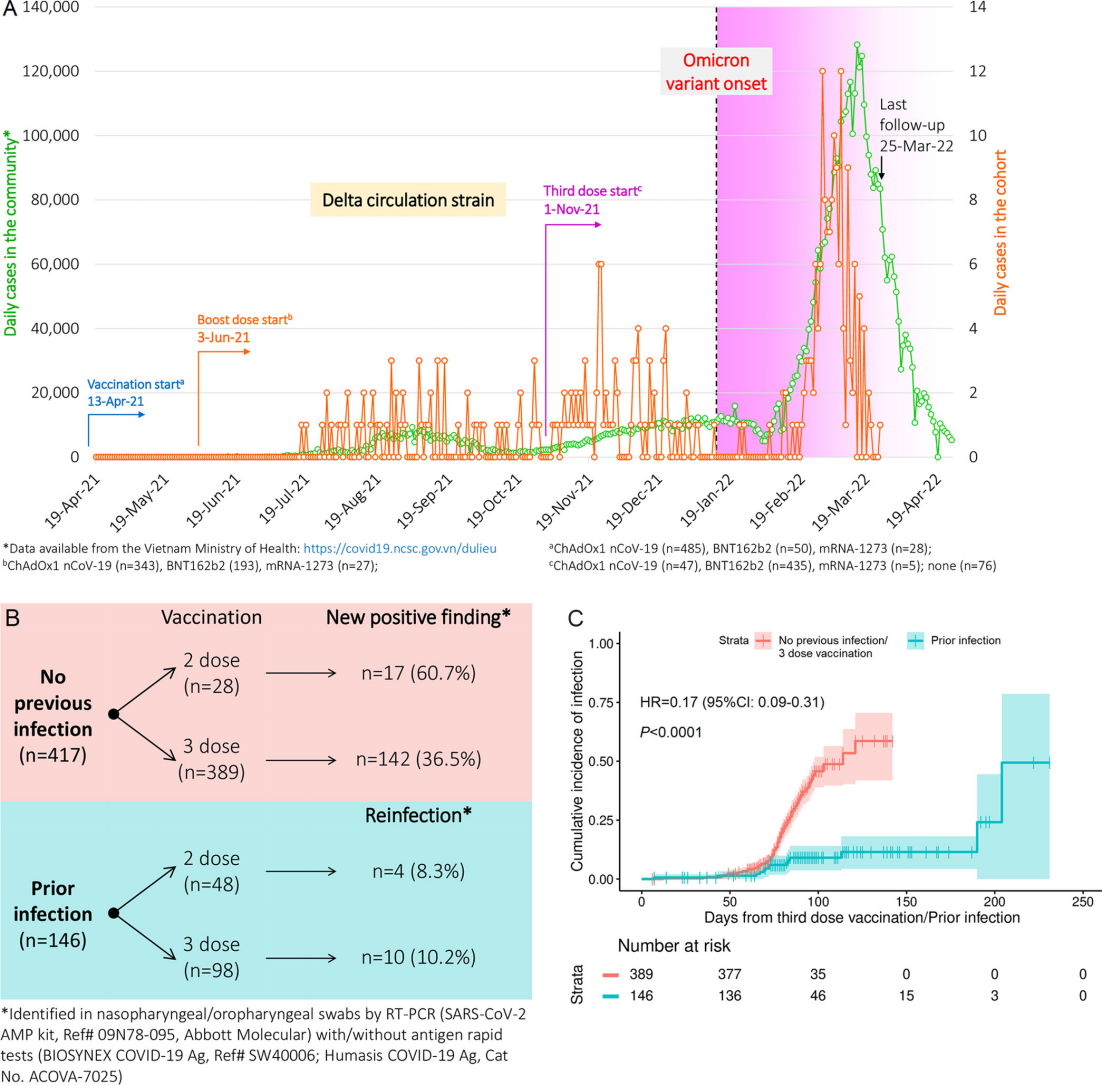
(A) Immunization timeline, follow-up, and daily infection numbers during pandemic waves. (B) Infection cases among groups. (C) Incidence of SARS-CoV-2 reinfection versus new positive findings in third-dose vaccination.

On 26-November 2021, the World Health Organization (WHO) designated the severe acute respiratory syndrome coronavirus (SARS-CoV-2) Omicron variant (B.1.1.529) as a variant of concern. In Vietnam, the first cases of the Omicron variant in the community were detected in Ho Chi Minh City on 19-January 2022 (notified by the Vietnam Ministry of Health, https://covid19.gov.vn/), following the Delta strain (1), and a wave of new infections followed soon thereafter (Fig. 1A). Similar to that of the Delta variant, the occurrence of the Omicron variant leads to immune escape caused by multiple mutations and thus decreases vaccine effectiveness (2). Remarkably, some evidence suggests that Omicron raises the risk of reinfection compared to previous waves (3–5). Here, we report the incidence of SARS-CoV-2 infection during the Omicron variant emergence in southern Vietnam among the preinfected and compare it with that observed in third-dose vaccination recipients.

This report is a part of the antibody response monitoring study at Cho Ray Hospital (approval number 1187 and 1298/GCN-HDDD), which included 563 adults (18 to 77 years) who completed a primary two-dose vaccination treatment from 29-April to 5-October 2021. The inclusion criteria were: vaccination with the ChAdOx1 nCoV-19 (AstraZeneca), BNT162b2 (Pfizer/BioNTech), or mRNA-1273 (Moderna) vaccines, according to the recommendations of the WHO; availability of prior test results for SARS-CoV-2 infection; and agreement to participate in the study by signing consent forms. Exclusion criteria include injection with different vaccines, loss of follow-up, or inadequate infection monitoring data.

After their primary vaccinations, 146 participants tested positive for SARS-CoV-2 (in the Delta period, 17-Jul-2021 to 12-Jan-2022), and 98 of them received a third dose after about 3 months of recovery (Table 1). 389 participants were negative with SARS-CoV-2 until the third-dose boost (from 1-November 2021 to 30-January 2022), while 28 others refused the injection. Reinfection was defined as a new positive result both after the Omicron variant occurrence (after 19-January 2022) and at least 90 days after the primary infection (6). The time interval was calculated as days from the first infection or the third-dose boost to the new positive result or last follow-up. Throughout the investigation, participants were tested routinely for SARS-CoV-2 infection biweekly or whenever a participant was exposed to a confirmed case or developed symptoms. We used the Cox proportional-hazards model to assess the relative incidence of infection between groups.

**TABLE 1:** Characteristics of included participants

Variable	Total (*n* = 563)	No previous infection (*n* = 417)		Prior infection (*n* = 146)		*P* value
		2 dose (*n* = 28)	3 dose (*n* = 389)	2 dose (*n* = 48)	3 dose (*n* = 98)	
Median age, yrs (95% CI)	40 (39, 41)	39 (32, 43)	40 (38, 41)	38 (34, 41)	39 (38, 42)	0.4415^*a*^ 0.5637^*b*^
Gender						
Female	217	9	150	18	40	0.8685^*a*^
Male	346	19	239	30	58	0.8054^*b*^
Occupation						
Nonhealthcare worker	100	10	71	9	10	0.0177^*a*^
Healthcare worker	463	18	318	39	88	0.1491^*b*^
Comorbitity						
No	448	21	310	40	77	0.8395^*a*^
Yes^*c*^	115	7	79	8	21	0.9089^*b*^

a Between four subgroups.

b Between no previous infection/3 dose and prior infection subjects.

c Including hypertension (*n* = 34): hypertension and chronic hepatitis/arthritis diseases (*n* = 2), chronic hepatitis (*n* = 11), arthritis diseases (*n* = 4), thyroid diseases (*n* = 16), respiratory diseases (*n* = 5), cardiovascular diseases (*n* = 9), diabetes (*n* = 12), diabetes and hypertension/chronic hepatitis (*n* = 5), dislipidemia (*n* = 4), vestibular disorder (*n* = 2), cervival diseases (*n* = 2), thrombocytopenia (*n* = 2), and others (*n* = 7).

By 25-March 2022 (median follow-up of 90 days, 95% confidence interval [CI] of 88 to 91 days), 173 persons (30.7%) tested positive for SARS-CoV-2 in the new pandemic wave (Fig. 1B), including 14 reinfections (9.6%) and 159 new positive findings (38.1%). Compared to those with no previous infection who received a third-dose vaccination, prior-infected subjects had a lower risk of the infection during the Omicron onslaught (hazard ratio [HR] = 0.17, *P* < 0.0001) (Fig. 1C). In a multivariable analysis, the protective role of a prior infection was still significant (HR = 0.19, 95% CI of 0.10 to 0.34, *P* < 0.0001), regardless of gender (*P* = 0.3297), occupation (health care/nonhealth care workers, *P* = 0.5339), and comorbidity status (*P* = 0.5586). Throughout the observation period (July 2021 to March 2022, 89.2% symptomatic and 10.8% asymptomatic cases), only one SARS-CoV-2 infected person with moderate symptoms required hospitalization without supplemental oxygen, and this person recovered 3 weeks later.

The limitations of this analysis are the small population studied and the lack of virus sequencing data for the determination of the Delta and Omicron variant samples.

Overall, in a prospective cohort from southern Vietnam during the onset of the Omicron variant, we noted that primary-vaccinated, prior-infected individuals have a lower incidence of SARS-CoV-2 infection compared to third-dose receivers. This finding is consistent with recent preliminary data (6).

### Data availability.

The data sets generated and/or analyzed during the current study are available from the corresponding author upon request.
